# Affective State during Physiotherapy and Its Analysis Using Machine Learning Methods

**DOI:** 10.3390/s21144853

**Published:** 2021-07-16

**Authors:** Patrycja Romaniszyn-Kania, Anita Pollak, Marcin D. Bugdol, Monika N. Bugdol, Damian Kania, Anna Mańka, Marta Danch-Wierzchowska, Andrzej W. Mitas

**Affiliations:** 1 Faculty of Biomedical Engineering, Silesian University of Technology, Roosevelta 40, 41-800 Zabrze, Poland; marcin.bugdol@polsl.pl (M.D.B.); monika.bugdol@polsl.pl (M.N.B.); anna.manka@polsl.pl (A.M.); marta.danch-wierzchowska@polsl.pl (M.D.-W.); andrzej.mitas@polsl.pl (A.W.M.); 2 Institute of Psychology, University of Silesia in Katowice, Bankowa 12, 40-007 Katowice, Poland; anita.pollak@us.edu.pl; 3 Institute of Physiotherapy and Health Sciences, The Jerzy Kukuczka Academy of Physical Education in Katowice, Mikołowska 72A, 40-065 Katowice, Poland; d.kania@awf.katowice.pl

**Keywords:** affective state analysis, electrodermal activity, emotional response, machine learning, signal analysis

## Abstract

Invasive or uncomfortable procedures especially during healthcare trigger emotions. Technological development of the equipment and systems for monitoring and recording psychophysiological functions enables continuous observation of changes to a situation responding to a situation. The presented study aimed to focus on the analysis of the individual’s affective state. The results reflect the excitation expressed by the subjects’ statements collected with psychological questionnaires. The research group consisted of 49 participants (22 women and 25 men). The measurement protocol included acquiring the electrodermal activity signal, cardiac signals, and accelerometric signals in three axes. Subjective measurements were acquired for affective state using the JAWS questionnaires, for cognitive skills the DST, and for verbal fluency the VFT. The physiological and psychological data were subjected to statistical analysis and then to a machine learning process using different features selection methods (JMI or PCA). The highest accuracy of the kNN classifier was achieved in combination with the JMI method (81.63%) concerning the division complying with the JAWS test results. The classification sensitivity and specificity were 85.71% and 71.43%.

## 1. Introduction

Effective disease management requires a constant search for factors relevant to improving the quality of care and patient safety [1,2]. Analysis of affective state refers to the way the person feels at any given time. The affective state consists of emotional responses, including experience, expression, and physiology. Those components aid in the interpretation or appraisal of the situation that provokes a given emotional response. It could be helpful in clinical practice to identify factors responsible for patient activation and engagement in the therapy [3]. Results of longitudinal studies suggest that patient activation predicts future health outcomes [4] and that it is possible to affect whether the patient successfully participates in the treatment and feels responsible for the healthcare process [5]. Emotions during healthcare are mainly triggered by invasive or frightening treatment experiences and pain during surgical procedures [6,7]. Technological development of the equipment and systems for monitoring and recording psychophysiological functions enables continuous observation of the changes in responding to a situation, making it possible to supplement the psychological data obtained through self-descriptive questionnaires with physiological ones [8,9,10].

According to classic theories, emotions, as complex ways of reacting, emerge in a multi-stage process [11,12]. Emotions are triggered when an event important for safety occurs. The response is formed due to the nervous system activation, cognitive assessment and behavioural reaction [13,14,15]. To evoke an emotion, physiological excitation has to be maintained to determine the situation significance for the individual [16]. According to Izard’s concept, four systems are responsible for emotion activation. They include the neural system, which is based on electric stimulation of the brain; the sensorimotor system, which activates the sensations through the body posture and facial expression; the motivation system in which emotions are generated as a result of sensory experiences and drives; and the cognitive system which applies to the categorization, assessment and comparison processes [11,12]. Russell and Carroll introduced differentiation of emotions based on arousal (presence or absence of stimulation) and valence (pleasure or displeasure) [17]. The arousal includes emotions on the biological level, in the form of physiological stimulation, while valence applies to the mental experiencing of emotions. The theory developed by van Katwyk et al. [18] includes, in addition to Russel and Carroll’s concept [17], the findings on the occurrence and course of a stress reaction [13,19]. In this approach, positive emotions with a high activation level are described as eustress, while negative emotions with a high-intensity degree are referred to as distress [18].

The previous results indicate the significance of neuroticism and negative emotions for intensifying stress experience during physiotherapeutic procedures in a non-clinical group [9,10]. Anxiety and stress are interrelated and condition one another [20,21,22]. Anxiety disturbs the correct rhythm of a person’s reaction and leads to exaggerated and inadequate reactions even to weak stimuli [23]. Persons characterized by a higher anxiety level tend to be shy, lack self-confidence, be reluctant to challenges, and not be able to cope with stress effectively. If persons with high anxiety factors are subject to assessment, they trigger many useless behaviours for fulfilling the task or even hinder it [24]. While negative emotions limit the number of cognitively available solutions to the problem, positive emotions extend the range of solutions [25,26,27]. Moreover, positive emotions play an essential role in building resistance to stress [28,29]. Experiencing negative affective states is also linked to low involvement in the action, which means that the persons devote less energy and reveal less will to undertake and continue the effort to complete the task and fulfil the requirements set to them [30]. According to Kahn’s assumptions, the persons who invest higher energy resources in doing a task have a broader perspective and are open to the possibilities and opportunities, which results in them facilitating other’s work [31].

In the biological approach, the emotions triggering is attributed to the amygdala [32]. It is responsible for the body’s defence reactions, stimulating the sympathetic nervous system [33]. The stimulation of sympathetic nerve fibres leads to the activation of eccrine glands, which immediately contributes to the changes in the skin conductivity and can be measured using the skin’s electrodermal activity (EDA) signal [34]. The EDA is one of the most often used signals for al and physiological analysis, as it represents both the prescriptive and pathological condition [35]. The sympathetic activity of the nervous system manages the cognitive and affective states, so the analysis of the EDA signal provides information about the autonomous emotion regulations [36]. The sympathetic nervous system stimulants affect the heart function as well—they improve conduction in the sinoatrial and atrioventricular nodes, which is reflected in such signals as the blood volume pulse (BVP) or heart rate (HR) values [37], being the second most commonly analyzed group of physiological data which characterize emotional states [38]. In addition, the acceleration signal indicates the subject’s movement, and the body or head movements can be used to detect emotions and their magnitude [39].

There is an increasing demand for finding solutions that support traditional assessment of the affective and cognitive states based on of the analysis of physiological signals (e.g., EDA, BVP, ECG or temperature) and employing machine or deep learning methods, which makes an independent application that predicts the patient’s condition and maximizes diagnosis accuracy [40,41].

The effectiveness of using machine learning methods and data from physiological signals (EDA), compared to the subjective assessment of depression was tested [8,42]. A multimodal approach was using similar bio-signals like in the presented article (BVP, EDA, EMG, temperature, and respiration) to develop a classifier to recognize negative emotions [43]. Another system based on ECG, EDA, skin temperature signals and SVM classifier were designed to assess emotions independently and reflect emotions on the autonomic nervous system [44]. An application has been developed using a photoplethysmographic and EDA signals recorded from a wearable device to analyse the user’s experienced emotions evoked by the presented affective images [45]. Repeated episodes of stress can disrupt a person’s physical and mental stability. Monitoring stress levels has therefore become an important and frequently addressed issue in recent years [40]. The stress level was assessed in a study using the Empatica E4 wristband and EDA signal analysis [46]. Efforts were made several times to predict the stress symptoms, the occurrence of anxiety or depression symptoms by using different machine-learning algorithms based on the results obtained with a complex DASS 42 questionnaire [47]. The effectiveness of the Matching Pursuit algorithm in recognizing emotions based on electrocardiogram and galvanic skin response signals while listening to music was also investigated [48] or during sleep to assess mental health [49]. In the study by Gou et al., the HRV signal determined from ECG, combined with SVM, enables recognizing states such as sadness, anger, fear, happiness, and relaxation [50]. The emotions were also evoked by visual and auditory stimuli (4 different videos) [51] and in addition, the ambient temperature was adjusted while watching the videos to enhance the experience. Based on features extracted from the recorded physiological signals (EEG, PPG, EDA), similar emotions as before—happy, relaxed, angsty, and sad—were recognised. Other studies have also proposed a system to recognize emotions (happiness, sadness, anger, fear) evoked by video recordings based on EDA signals combined with different classifiers—gradient-boosting decision tree, logistic regression, and random forest [52]. The differentiation of six other states (joy, sadness, fear, disgust, neutrality, and amusement) based on physiological signals was also the subject of an analysis based, among other things, on skin temperature (SKT), skin conductance (SKC), blood volume pulse (BVP) and heart rate (HR) [53]. Subsequent studies attempted to distinguish between another set of emotions—sadness, anger, stress and surprise, based on short fragments of the electrocardiogram signal, skin temperature variation, electrodermal activity and classification by machine learning techniques [44]. A tool developed by a team led by Carpenter [54] is an example of another screening tool constructed using machine learning to evaluate the risk of anxiety disorders occurrence. Physiological signals were often used to analyse emotional states because they represented the most authentic response of the organism, which is beyond human control. Therefore, in combination with machine learning or deep learning, high accuracies were achieved when distinguishing between individual emotions [55,56]. The paper by Wei et al. proposes a different classification method than previously presented, the Weight Fusion strategy, for the recognition of emotional states based on signals such as electroencephalography (EEG), electrocardiogram (ECG), respiration amplitude (RA) and electrodermal activity (EDA) [57]. The paper by Pinto et al. demonstrated a qualitative approach to the analysis of emotions based on physiological signals (ECG, EDA, EMG) [58]. They investigated whether and which signals carry more information in emotional state recognition systems. The general broad interest in methods of using artificial intelligence is reflected not only in assessments of emotional states but also in direct support of medical science for example to improve the performance of conventional contactless methods for heart rate measurement [59] or to detect and classify cardiac arrhythmia [60].

There were also a lot of different approaches to emotion assessment based on data other than physiological signals from wearable devices. Several methods used neuroimaging combined with machine learning algorithms to objectivize psychological diagnostics of patients [61,62] and search for the biomarkers that identify the particular emotional state [63]. Another approach was for systems to recognise emotions based on facial expression [64,65,66,67] or facial micromovements [67]. However, in each of the systems mentioned above, no attempt was made to objectify the emotions experienced, as in each case, the label of the emotional state was merely the subjective evaluation of the subject induced intentionally by a particular activity. The problem remained in recent research and developments. Emotion recognition using Convolutional Neural Networks (CNN) or other deep learning methods (like ResNet, LSTM) was based on the signals recorded when these emotions were evoked [68]. The two largest learning databases, DEAP [69] and MAHNOB-HCI [70], were collected during an experiment where the patients’ physiological signals were recorded when they were watching images or videos intended to evoke specific emotions. The problem of objectifying judgments independent of the subject is a problem that has been studied in many fields, but in the case of artificial intelligence, these classifiers were based on learning sets containing emotions that were intentionally elicited in the subject [71,72].

To the best of the author’s knowledge, there is a gap in identifying the affective states that occur during physiotherapeutic procedures and the description of their significance for an individual’s actions results. Based on the patient’s visual observation during rehabilitation, it is hard to unambiguously determine if the implemented therapy maximizes the effects at the patient’s moderate own effort. This study focuses on the analysis of the individual’s affective state. The results reflect the excitation expressed by the subjects’ statements acquired with psychological questionnaires. They are also expressed by the values of the characteristics determined based on physiological signals. The changes in the motivation to take action are another effect of the excitation. They are significant for the levels of energy, enthusiasm, and concentration on the fulfilled tasks [73]. A reduced effort in the exercise, doing the exercise slower, or ignoring the therapist’s recommendations can manifest the consequences of a lack of involvement resulting from low intensity of emotions or prevalence of negative emotions.

## 2. Materials and Methods

### 2.1. Research Group

The research group consisted of 49 students (22 women and 25 men) of the Jerzy Kukuczka Academy of Physical Education in Katowice aged 19–26. Individuals with a current medical condition, current medication intake, or lifetime history of any neurological or psychiatric disorders were excluded from the experiment. All participants were provided written informed consent before the beginning of testing. The Bioethics Committee of the Jerzy Kukuczka Academy of Physical Education in Katowice approved the study protocol (No. 3/2019) and conducted it according to the Declaration of Helsinki.

### 2.2. Study Design

The research was carried out in a laboratory of the Institute of Physiotherapy and Health Science of the Jerzy Kukuczka Academy of Psychical Education in Katowice in two interconnecting rooms. The rooms were adapted to the research requirements, creating a functional and comfortable space. One room was dedicated to welcome the volunteers and fill out the consent for research participation. The second room had exercise areas and was adjusted to take subjective and sensor-based measurements while remaining private.

**Figure 1:**
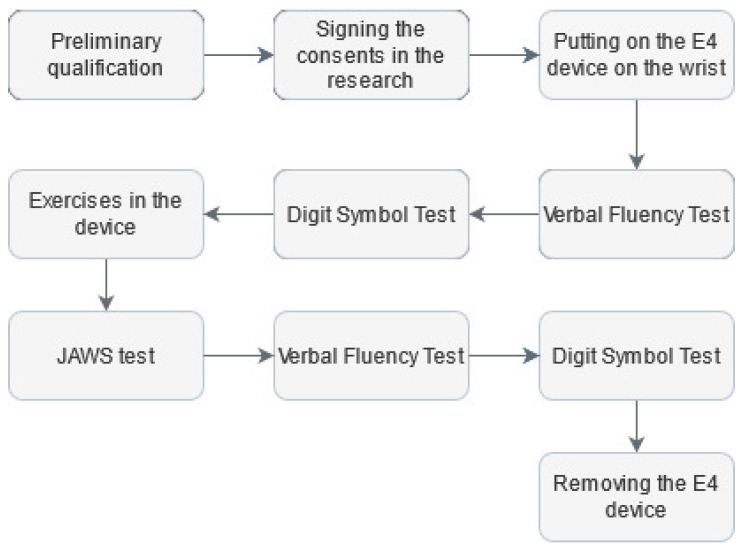
Research protocol.

The measurement protocol (Figure 1) was based on a previously proposed concept [9]. This approach used both quantitative and qualitative assessments.

To record physiological signals and continuously monitor the participants during the research, an FDA-approved Empatica E4 wristband was placed on the wrist of the non-dominant hand [74]. Then, to assess executive abilities, the participant was exposed to the Verbal Fluency Test (VFT) in the phonetic version [75]. The VFT is often used in neuropsychology, both in clinical and experimental assessment, due to its performance simplicity. Its correct performance depends on executive functions efficiency, working memory and linguistic resources in long-term memory [76]. Next, the Digit Symbol Test (DTS) was used to assess cognitive functions, including learning, maintaining attention, and solving tasks [77]. After completing the above tests, each person was asked to go to a room where a prototype of the Disc4Spine (D4S) diagnostic and therapeutic system for supporting the rehabilitation of postural defects was located. The standing exercise module is a therapeutic device for corrective exercises involving single-step short muscles responsible for motor control [78]. Immediately after leaving the D4S, the subject was requested to fill out scales assessing their affective state during exercise activity by the study-adapted measure. Original Job-Related Affective Well-Being Scale (JAWS) measures the varied affective responses to perceived job conditions and outcomes, consisting of modified instructions to place them in the research context [18]. The new version was as follows: “Please use the following scale to answer the question: how often do you feel the following during the exercises?” The study used a 12-item version. It comprises 12 various emotions, both positive and negative. The responses format was a 5-point Likert scale from (1 = never, 2 = rarely, 3 = sometimes, 4 = often, 5 = ery often).

The final elements of the research protocol were to retake the VFT for a different letter than at the start of the study and retake the DST. After that, the Empatica E4 wristband was dismounted.

### 2.3. Data Acquisition

The first device used was the Empatica E4. It consists of a thermometer and a photoplethysmographic sensor measuring cardiac signals such as blood volume pulse (BVP), from which heart rate (HR) and inter-beats interval (IBI) are then computed. Each HR sample was averaged over the previous ten seconds. An electrodermal activity (EDA) sensor measured changes in the skin electrical conductivity, and a triaxial accelerometer (ACC X/Y/Z) monitored the patient’s activity during the tests. The BVP signal was recorded at 64 Hz, and the HR signal was sampled at 1 Hz, EDA, and temperature—at 4 Hz, and acceleration at 32 Hz. In the Verbal Fluency Test, the subject’s goal was to generate within 60 s as many words as possible in the native language, which start with a given letter, each time chosen randomly (excluding X, Y, and all Polish diacritic characters). A proprietary mobile application was used to count the number of uttered words, whereas the leading letter and the remaining time were continuously presented. The participant received a sheet of paper on which there were two lines in the upper part to perform the DST—the first line contained consecutive numbers from 1 to 9, and the second, lower line, included symbols corresponding to those numbers. The task was to write down as many symbols as possible corresponding to consecutive numbers from the range 1–9, given at random, within 60 s. In the D4S module, while the research participant was correctly positioned and secured, they were asked to perform three consecutive exercises of varying difficulty activating muscles under the physiotherapist’s supervision. The first exercise consisted of alternating forward and backward pelvic tilt for 60 s at a frequency of one sequence per second. Then the subject was asked to perform the external rotation of feet for 10 s with maximal force, which was measured by a resistance bar that was a component of the system. The third exercise consisted of the following sequences: external rotation of the feet, external rotation of the knees, anterior tilting of the pelvis, shoulder retraction, and, finally, elongation of the spine and holding this position for 10 s. In the next step, the paper version of the JAWS questionnaire was filled.

## 3. Data Analysis

**Figure 2:**
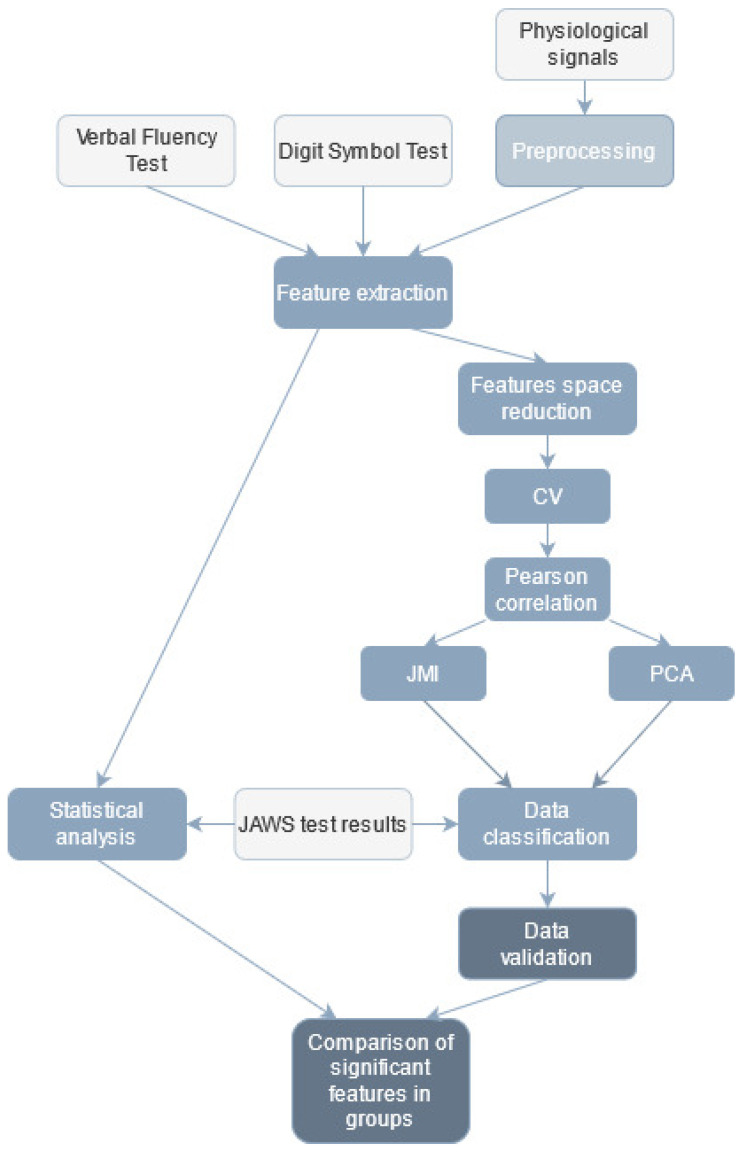
Workflow of data analysis (light grey—input data, medium grey—data classification, dark grey—data validation).

The workflow of data analysis is presented in Figure 2.

The research data were combined and constituted one extensive database of coefficients subjected to further analysis.

### 3.1. Executive and Cognitive Abilities

The analysis of the Verbal Fluency Test results (before and after exercise, independently) was focused on the number of spoken words, the mean time between utterances, as well as the popularity of a given letter in the Polish language according to the National Corpus of Polish [79] and the author’s fluency coefficient defined as the quotient of the number of uttered words and the letter popularity. Additionally, the differences between the number of spoken words, the mean time, the letter popularity, the fluency coefficients before and after therapy were calculated. In total, 12 features were obtained. A dedicated application was used to facilitate the VFT subsequent analysis, which generated a .csv file with the results after the test was performed. The differences between the coefficients mentioned above for the test performed before and after the exercises were also analyzed. These results were the essential piece of information, as they indicated verbal-letter fluency.

Based on of the Digit Symbol Test results analysis, a total number of nine features (before and after exercising, independently) were determined, such as the number of matches, the number of correct matches, the author’s match rate (calculated as the quotient of the number of correct to all matches). Furthermore, the differences between values obtained before and after exercises of the following coefficients were calculated: the number of all matches, the number of correct matches, the difference in match rates. In this DST version, the participant’s most crucial task was to match as many number-symbol pairs correctly as possible. It was also important to remember that they had to do it one by one, according to the given sequence of digits. To evaluate the test, a manual analysis was carried out.

### 3.2. Psychological Measurement

The intensity of current feelings as a reaction to the research situation by computing the sum of the answers of 12 items was assessed. The calculations were conducted in PRO IMAGO 7.0. The internal reliability of the scale measured with Cronbach’s α was 0.85. The cut-off for dividing the research group is the median score (value 44), assuring the distribution-independent division. There were 15 people in the below-median group and 34 in the above-median group.

### 3.3. Signal Preprocessing

**Figure 3:**
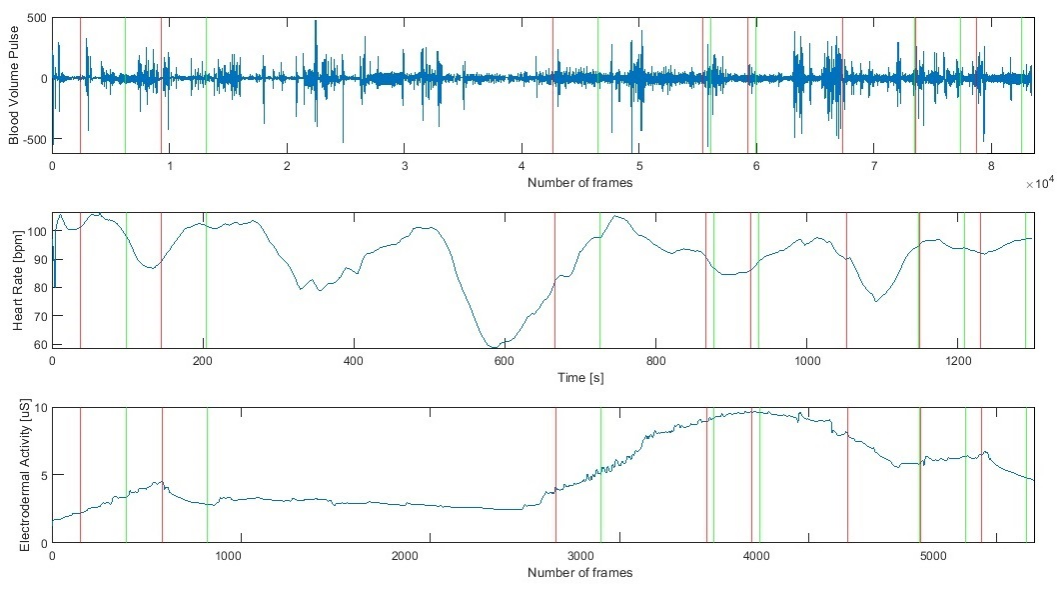
Cardiac (Heart Rate, Blood Volume Pulse) and Electrodermal Activity raw signals during successive stages of the research protocol (a red line indicates the beginning and a green line the end of a stage).

The Empatica E4 wristband allows recording the time markers indicating the beginning of the occurrence of a given protocol element (Figure 3). On this basis, each of the analysed signals was divided into time segments such as:

- VFT before exercise (vft1),
- DST before exercise (dst1),
- exercise no 1 (ex1),
- exercise no 2 (ex2),
- exercise no 3 (ex3),
- psychological test (ptest),
- VFT after exercise (vft2),
- DST after exercise (dst2).

For each signal, features were determined from the individuals’ time intervals. Finally, from all modalities, 1133 features were extracted.

#### 3.3.1. Heart Signals

Empatica E4 captured the Blood Volume Pulse signal. The HR signal was determined from the BVP by the algorithm proposed by the Empatica wristband designers. The analysis method was the same for both BVP and HR and involved short-term signal fragments and the determination of statistical and entropy-based features [80,81]. Due to the characteristics of the HR signal, it was not filtered before further calculations. The following statistical parameters were evaluated for each segment in every signal: mean, median, mode, 25th, and 75th percentile, quartile deviation, minimum and maximum value, variance, the fourth and fifth-order moments, skewness, kurtosis, root mean square, range, and the total sum of values. In addition, the following entropy-based features were determined for each of the fragments: total energy, mean and median energy of the signal, and entropy. In total, 20 parameters were calculated for each signal segment.

#### 3.3.2. EDA

The next signal to be analyzed, recorded using Empatica E4, was the electrodermal activity. A wavelet transformation from the Symlet wavelet family was used to filter the signal [82]. The maximum value of the decomposition level was set according to Equation (1).

(1) $$dec\_level=\left\{\begin{array}{cc} log_{2}n & log_{2}n<10\\ 10 & log_{2}n\geq 10 \end{array}\right.$$

Based on the available literature [9,10,34,35,81], for each part of the EDA, the same statistical parameters as for the heart signals were computed. Furthermore, the following coefficients were also computed from the EDA signal: total energy, mean and median energy of the signal, entropy, coefficient of the slope of the regression line allowing to determine the trend—tonicity of signal, coefficients of regression line shift, a distance of values of consecutive EDA samples from the regression line, number of signal intersections with the regression line and the quadratic metric of the discrepancy between predicted and observed data (obj). Characteristic parameters were also determined from the EDA signal. The first of them was Galvanic Skin Response (GSR), which presents sudden changes in skin electrical resistance caused by momentary emotional stimulation, increasing the sympathetic nervous system activity [34]. On this basis, the number of GSR responses per minute—rpm, their energy, and the number and energy of significant GSR, i.e., those with a value above 1.5 uS, were computed. In total, 264 features–33 features per stage—were determined for the whole EDA signal considering the individual steps.

#### 3.3.3. ACC

The acceleration was recorded in three axes—X, Y, and Z using the E4 wristband. The ACC signal was analyzed independently for each axis, according to the same scheme. The first step was to divide the signal into time segments. Then, for each segment, statistical parameters were calculated: mean, median, variance, 25th and 75th percentile, quartile deviation, minimum and maximum value, signal range, the fourth and fifth-order moments, skewness, kurtosis, RMS, total sum, mean and median of the ACC signal energy. For the ACC signal, 20 features were determined for each stage, for a total of 160 features for each axis.

### 3.4. Statistical Analysis

The results of the JAWS (jw) tests were converted to dichotomous factor variables according to the following criteria:

(2) $$\mathrm{JAWS}=\left\{\begin{array}{cc} 0 & jw<44\\ 1 & jw\geq 44 \end{array}\right.$$

Next, all the computed variables, which were all quantitative, were compared according to the JAWS division using the Student’s *t*-test for independent samples, the Welch’s *t*-test, or the U Mann-Whitney test, depending on which assumptions were fulfilled. They were verified using the Shapiro-Wilk test (normality), the F-test (homogeneity of variances), and the chi-squared test (equal sample sizes). The significance level alpha was set to 0.05 for each test.

Additionally, based on the distribution presented above (Equation (2)), the statistical significance of the difference of the JAWS test results between groups was tested with the Shapiro-Wilk W test (normality of distribution) and Mann-Whitney test (equality of medians), at an alpha significance level of 0.05 for each test.

### 3.5. Features Selection

**Figure 4:**
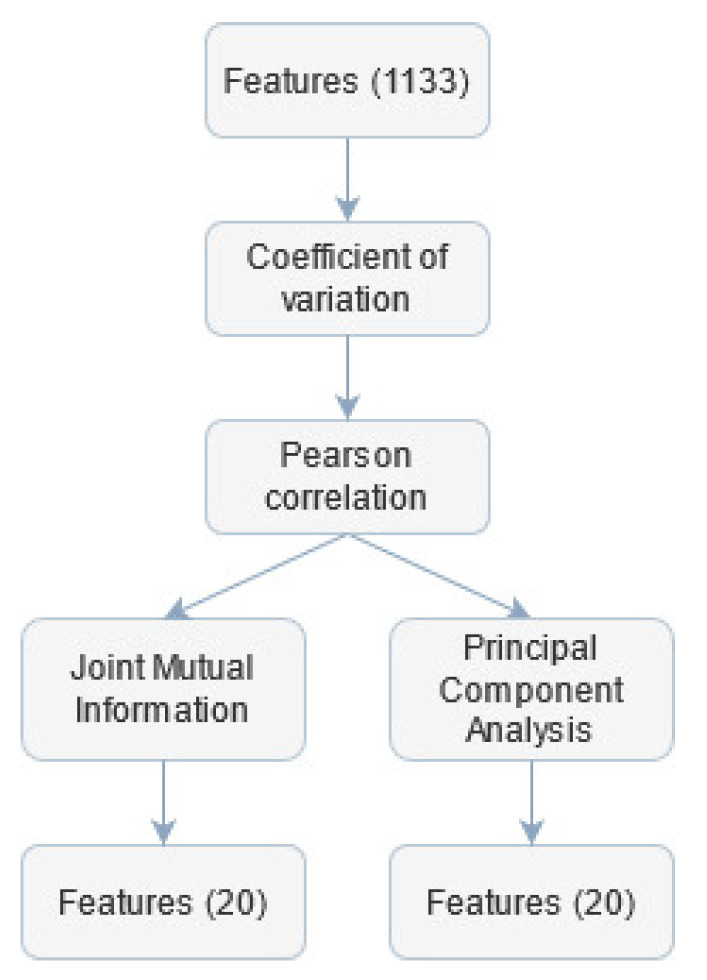
Subsequent steps of feature selection.

The parameters extraction stage provided a set of far too numerous features to avoid classification overfitting problems (Figure 4). Therefore features selection was performed to decrease the classifier test error.

First, the coefficient of variation ($c_{v}$) for each feature was calculated. If the value of $c_{v}$ for the given parameter was lower than 0.02, this feature was considered quasi-constant and removed from further analysis. The threshold for the $c_{v}$ was set to such a low value to avoid rejecting informative coefficients.

Next, the Pearson correlation matrix was calculated. Features for which this coefficient was greater than 0.98 were labelled as candidates for being removed. Among this set, the feature was found, which was over-threshold correlated with the largest number of other variables and was then excluded from the final set. This procedure was repeated until there were no over-correlated coefficients. The threshold value was chosen based on other work concerning similar topics [83]. The computed features had different ranges, so there was a need to rescale them before employing further analysis. This step was necessary because feature selection and classification algorithms can be sensitive to extreme values. The rescaling was performed using min-max normalization. To discriminate features with small differences, the transformation of features values was proposed, according to exponential function with base equal to 1.1. This value was chosen empirically—such transformation was searched to strengthen the differentiation between values within one feature.

Furthermore, a non-linear Joint Mutual Information (JMI) optimization method was used. It focuses on increasing the complementary information between features using a minimum/maximum criterion to solve the information overestimation problem [84]. The first 20 features with the highest JMI values were selected for further classification independently from class division, according to JAWS.

Another approach to select features was employed—the Principal Component Analysis (PCA) method. The PCA is an unsupervised dimensionality reduction technique that analyses correlations between different features based on their linear combination and reduces the number of variables while maximizing their variability [85]. In the presented research, the input data were transformed using the PCA into a new feature space, and the 20 most differentiating variables were selected.

### 3.6. Classification

For classification purposes, the k-Nearest Neighbours (kNN) classifier was used in supervised learning [86]. The supervised classification task was to estimate the label of a given feature vector based on data distribution. In the presented approach, the value of k was set empirically to 10, and for the similarity evaluations, the cosine metric was employed. Other metrics were also tested, but the achieved results were less differentiating. It was decided to choose machine learning (ML) over deep learning (DL) methods because in DL, the datasets are based on whole signal waveforms, divided into frames, and recorded over a longer time. In the presented work, due to the short time segments (30–60 s), only features for individual stages were extracted from the analysed signals. The research presented here is carried out in laboratory conditions using the prototype of the D4S device, which means that we are dealing with a simulation of short therapy sessions. Emotional changes, both psychological and physiological, occurring in the patient’s body during such exercises are not as fast-changing as in studies/experiments analysing emotions of everyday life. The patient will not experience such intense and sudden emotional stimuli as, for example, when receiving tragic information because our situation is related to regular physical exercise. The therapy of people with scoliosis is a long-term process that does not provide momentary strong pain stimuli. Hence, fatigue has different characteristics than in random situations. In the choice of optimisation (PCA and JMI) and classification methods presented above, less complex approaches were chosen due to the work’s interdisciplinary nature. The way these methods work and the results obtained are understandable for psychologists and physiotherapists who are members of the authors’ team.

### 3.7. Method Validation

The basic characteristics of classification, indicating the accuracy and quality of the classifier performance were calculated, i.e., accuracy (ACC), sensitivity (TPR, True Positive Rate), specificity (TNR, True Negative Rate), precision (PPV, the number of positive class predictions that belong to the positive class) and F1-score (the harmonic mean between precision and sensitivity) according to the following formulas:

(3) $$ACC=\frac{TP+TN}{TP+TN+FP+FN}$$

(4) $$TPR=\frac{TP}{TP+FN}$$

(5) $$TNR=\frac{TN}{TN+FP}$$

(6) $$PPV=\frac{TP}{TP+FP}$$

(7) $$F_{1}=\frac{2*TPR*PPV}{TPR+PPV}$$

True Positive (TP) values are the number of input feature observations classified as positive by the kNN classifier. False Negative (FN) refers to positive observations classified in the negative group. Whereas the True Negative (TN) is the real negative samples, and False Positive (FP) refers to the negative group positive observations. The above coefficients were based on the study group model division into positive/negative classes to the JAWS test results.

## 4. Results

### 4.1. Statistical Analysis of the Signals

For each signal Table 1 shows the lists of the statistically significant features, indicating from which stage of the research protocol they came.

The greatest number of statistically significant features was determined for the ACC X signal to 34 features. The ACC Y signal was also characterized by many statistically significant coefficients (27 features). The ACC Z and the BVP and HR cardiac signals exposed a small number of statistically significant features. The EDA signal had only one significant feature.

### 4.2. Psychological Measurement

Due to the non-normal distribution of the JAWS values in the two groups ((W(49)=0.956,p=0.063)), the analysis of differences between those groups (below the median and above the median) was computed using the Mann-Whitney U-test. Descriptive statistics for each group are shown in Table 2.

It is found that the intensity of current feelings was significantly higher (U(49)=510.00,p<0.001,Cohen’s d=1) in the group above the median (M = 47.92) than in the group below the median (M = 40.00). It shows that the experiences of affective states were characterized by higher frequency and intensity of emotions. The less intensive reaction to the stimuli, characteristic for the group below the median, in behavioural categories, can be described as restraining from the spontaneous expression of emotions, and consequently gestures and behaviour. A more detailed analysis of the differences in the intensity of individual emotions is presented in Table 3.

Results show that such emotions as gloomy, energetic, inspired, relaxed, and satisfied are more intensive of the group above the median. Cohen’s d value indicates a medium to large effect size (Cohen’s 0.363<d<0.555). The state described as at ease intensity is less for the group above the median (Cohen’s d=0.508). There are no differences between each group for emotions like anger, anxiety, discouragement, disgust, excitement, and fatigue.

### 4.3. Executive and Cognitive Abilities

Table 4 presents the mean values of the individual features computed for the VFT and DST before and after exercising.

**Figure 5:**
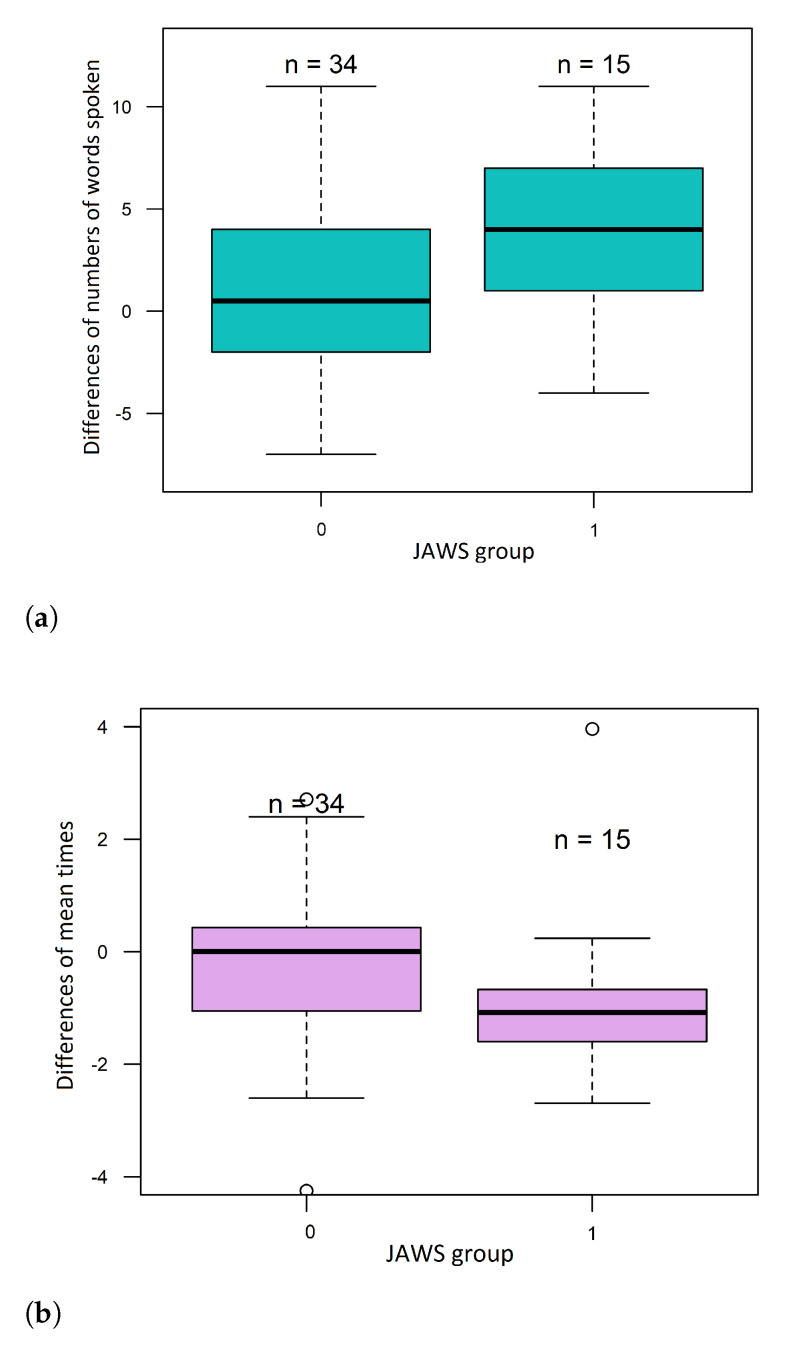
Boxplots of (**a**) the numbers of differences of words spoken and (**b**) the differences in mean times between words.

It can be noted that there was an increase in the number of words spoken by the subject. This difference was statistically significant according to the JAWS division. Subjects from JAWS group 1 had a significantly higher median difference of words spoken than group 0–four words more after exercising than before, whereas patients from group JAWS 0–0.5 words more after exercising than before (Figure 5a).

The mean time between consecutive spoken words decreased. This difference was also statistically significant according to the JAWS division. The mean time in JAWS 1 subjects decreased by 1.1 [s] (median difference in the mean time), while in the JAWS 0 group, the mean time did not change—the median difference was 0.01 [s] (Figure 5b). The popularity of chosen letters in the second VFT was lower than in the first approach. However, this difference was not statistically significant. The mean value of the fluency coefficient also changed. It was higher in the VFT after the exercises than before but without statistically significant differences.

In the second DST attempt, all obtained parameters increased. The number of total matches increased by about six number-symbol pairs, the accuracy of matching symbols to numbers was also improved as well as the digit rate. However, none of the mentioned features showed statistical significance against the JAWS division.

### 4.4. Data Classification

#### 4.4.1. Features Selection

Based on the value of $c_{v}$ and the correlation coefficient, 636 features were selected for further analysis. The JMI algorithm provided the JMI coefficient value for each trait. 20 features with the highest JMI values were selected, with a cut-off limit of 5.485623, ranging from 0 to 5.6147.

The selected features are presented in Table 5.

The second approach to optimizing the number of features was the PCA transformation. The number of components was limited to a maximum of 20 of those with the highest variance.

#### 4.4.2. Machine Learning

Table 6 presents the kNN classifier performance for 20 features selected independently with the JMI and PCA algorithms for the division into two classes relative to the JAWS labels.

The highest classifier accuracy was achieved for the kNN in combination with JMI (81.63%). The highest sensitivity (TPR) was achieved for the kNN with PCA. However, for the same combination, a low specificity was obtained, reflecting a low accuracy of the classifier compared to kNN with JMI. The $F_{1}$-score, which shows the harmony between the precision and sensitivity values, reached its highest value (0.90) for kNN with JMI.

Overall, the kNN classifier presented the best performance in combination with JMI—it achieved the highest accuracy, precision, $F_{1}$-score, and sensitivity value was comparable to specificity. The PCA method in combination with kNN resulted in slightly lower results but still satisfactory.

## 5. Discussion

Physiotherapy as a sum of medical influences on the patient, whose fundamental objective is to restore the highest functional performance possible [87], can be strengthened by monitoring the affective state that reflects the attitude to the effort. The information for the therapists on the emotions experienced by patients can promote relationship building and strengthen [88], and support coordination of actions [73], similarly to the affective contagion phenomenon [89].

The protocol presented in this paper was based on the acquisition of the electrodermal activity signal, cardiac signals, and accelerometric signals in three axes. Subjective assessments were gathered using questionnaires that measured the intensity and diversification of emotions (JAWS), cognitive skills (learning, attention focusing and shifting, processing speed—DST), and verbal fluency (VFT). The physiological and psychological data were subjected to statistical analysis and then to a machine-learning process using different feature selection methods (JMI or PCA). In the JMI method, the twenty most promising features were selected, but they were only BVP and EDA signal features. It is a likely confirmation of the significance of both those signals in the psychophysiological analysis to the sympathetic nervous system stimulation and experiencing affective states. Comparing the statistically significant features and those directly indicated as the differentiating ones with the JMI method, it should be noted that only one was selected in both cases. Hence, a conclusion can be drawn that some features indicated as statistically significant ones are the differentiating features for machine learning methods at the same time. The highest accuracy of the kNN classifier employed in this study was achieved in combination with JMI (81.63%). The classification sensitivity and specificity were 85.71% and 71.43%, respectively, which concludes that most physiological observations were classified correctly according to the results of psychological analyses. Both factors are essential in medical examinations because they are the evidence of the correct diagnosis [90]. The factors in this paper determined the classification of the subjects’ intensity and diversification of the emotions experienced.

Psychological measurement tests enable measuring emotions directly after they were induced rather than during their occurrence [91]. The use of measurement sensors can take over the function by continuous measurement of physiological reactions. It also helps to avoid referring to the subjects’ retrospective, which matters when emotions are measured because humans tend to overestimate the frequency and intensity of the emotions they experience when they evaluate the emotions retrospectively [92]. Based on the obtained results, it should be noted that BVP and EDA signals are objective measures supporting the psychological assessment. It offers the opportunity to support behavioural diagnostic and analyze the subjects’ physiological reactions in various everyday situations more thoroughly. The approach based on the EDA signal analysis and its features is an example of such attempts [10]. To objectivize the patient’s emotional state during therapy, a set of analytical parameters—signal errors was proposed, based on which the affect classification accuracy reached the level of 86.37%.

There are papers on similar topics based on an analysis of physiological data correlated with psychological data that evaluate psychic disturbances, such as depression or anxiety states. Ghandeharioun et al., in their analyses, by using different machine-learning methods - Random Forest and adaptive boosting (AdaBoost) obtained a low RMSE (Root Mean Square Error) value. It concluded that depression symptoms could be measured continuously using data from sensors [8]. An analogy can be discovered between the studies mentioned by Ghandeharioun and those presented in this paper. Both analyses were based on a subjective assessment of psychological tests, and the studies aimed to objectivize the behavioural state based on physiological data. In the paper authored by Richter et al., the STAI questionnaire was used to evaluate anxiety and depression levels [42]. The data obtained from a set of behavioural tests (from a ready-made database) and their analysis through machine learning methods—Random Forest algorithm—helped to detect unique patterns characterizing depression and anxiety states at the maximum accuracy of 74.18%. The key cognitive mechanisms in anxiety and depression were also indicated, but the whole research protocol was based on subjective data from psychological tests. The research was not objectified using physiological data, which can significantly affect the results.

Carpenter et al. proposed a screening tool for assessing the risk of anxiety disorders in children based on the Preschool Psychiatric Assessment (PAPA) test and machine learning algorithm [54]. They used the Alternating decision trees classifier (ADtrees) and data from a public database. The works revealed that machine learning use helped to reduce the number of items necessary to identify anxiety disorders in children by one order of magnitude with 96% accuracy. Still, it can be noticed that this is a subjective assessment based only on psychological data not supported by any physiological data. In the paper by Priya et al., the predictions concerning anxiety, depression, and stress were made using different machine learning algorithms [47]. The psychological data originated from the Depression, Anxiety and Stress Scale (DASS 21). The best results for the above-mentioned emotional states predictions were achieved with the Random Forest classifier—79.80% for depression states. The kNN method was also employed, but it did not render satisfactory results. The $F_{1}$ score factor was revealed to be an essential parameter for such algorithms, combined with behavioural data, while the specificity parameter revealed that the algorithms were susceptible to negative results. In the studies presented in this paper, there is an analogy with regard to the evaluated behavioural states and the applied classification methods, with such an advantage that the classification accuracy achieved by Priya et al. with the kNN method for anxiety reached 69.8%, which is a worse result compared to the results obtained in this paper, i.e., 77.5%. Moreover, only subjective questionnaire data, with no objectivisation attempt, were used. A different approach to emotional state assessment was presented in a paper authored by Boeke et al. [63]. The authors sought biomarkers that describe the patient’s mental condition based on neuroimaging combined with machine-learning algorithms. Still, no significant biomarkers were found. The cost of neuroimaging test and its availability, compared to easy monitoring of the patient through continuous recording of physiological signals, is another uncertainty.

In the D4S system, chronically ill people with scoliosis will be subjected to therapy. Their emotions will mainly depend on their character and not on the momentary state induced by the exercise performed with the therapist. We assume that the research carried out in the future will make it possible to determine the behavioural profile of the patient and later will allow us to develop a system for the physiological and behavioural assessment of the patient during the therapy. The laboratory conditions in the presented research protocol were chosen deliberately because the D4S system will be used in therapeutic sessions when the physiotherapist is working with patients and not during everyday activities. Our further work will be focused on extending the presented classification with real-time signal analysis, which could improve the therapy and allows to alert the therapist and make changes on spot.

## 6. Conclusions

In affective states accompanied by a high degree of excitation, a clear emotional expression is observed. Psychological measurement can provide data on the intensity and diversification of emotional reactions, which detection is possible by measuring selected physiological signals. The EDA and BVP signals demonstrate the highest dependence of the behavioural state on the physiological state. The tested protocol can be a part of a system, oriented towards equipping both the patient and the professional with the tools to manage diseases like scoliosis. The analysis of the patient’s functioning during the therapy offers the possibility of developing interventions that will be aimed at controlling and regulating the experienced emotions to a level that will help the patient to recover. In the approach of applying behavioural medicine to rehabilitation, the therapist should systematically consider the biopsychosocial factors that are relevant to the patient’s activity, both temporarily and, what is more important, in the long term.

## Figures and Tables

**Table 1:** Statistically significant features according to JAWS division, signal analysed, and protocol stage.

Signal	Sigificant Features
BVP	total sume (vft1), kurtosis (dst1), minimum (ex3), maximum (ex3), 4th moment (vft2), 5th moment (vft2), skewness (vft2), RMS (vft2)
HR	sume power (vft1), mean (ex1), 25 percentile (ex3), skewness (ex3), median (ptest), variance (ptest), minimum (dst2)
EDA	5th moment (vft2)
ACC X	mean (vft1, ex2, ex3, dst2), median (vft1, ex2, ex3, ptest), variance (dst1), 25 percentile (vft1, ptest, vft2), 75 percentile (vft1, dst1, ptest, vft2, dst2), minimum (vft1, dst1, ex2, ex3, ptest, vft2, dst2), maximum (vft1, dst1, ex2, ex3, ptest, vft2), range (dst1), 4th moment (dst1), 5th moment (ptest), total sume (ex2)
ACC Y	mean (vft1, ptest), median (vft1), 25 percentile (vft1, dst1, ptest), 75 percentile (vft1, dst1, ex2, ptest), minimum (vft1, dst1, ex1, ex2, ex3, ptest), maximum (vft1, dst1, ex1, ex2, ex3, ptest), total sume (vft1, ex1, ptest), range (dst1), kurtosis (vft2)
ACC Z	median power (dst1), 4th moment (dst1), skewness (ptest), kurtosis (dst2)

**Table 2:** Descriptive statistics of psychological data.

Variable	JAWS	
	below median	above median
Mean	35.25	46.88
Standard deviation	2.98	3.06
Median	37.60	46
Min	33	45
Max	41	57
Range	12–60	

**Table 3:** The intensity of individual emotions measured by the JAWS test.

	Group	Mean	SD	Statistics	*p*	Effect Size
JAWS1 angry	below	4.533	0.915	256.500	0.978	
	above	4.676	0.535			
JAWS2 anxious	below	4.200	1.082	270.500	0.717	
	above	4.441	0.660			
JAWS3 at ease	below	2.400	1.056	416.000	0.001	0.631
	above	3.618	0.922			
JAWS4 gloomy	below	4.133	1.060	339.000	0.022	0.329
	above	4.794	0.410			
JAWS5 discouraged	below	4.000	1.309	276.500	0.619	
	above	4.382	0.652			
JAWS6 disgusted	below	4.467	0.915	271.500	0.662	
	above	4.588	0.743			
JAWS7 energetic	below	1.867	0.990	462.00	<0.001	0.812
	above	3.882	1.008			
JAWS8 excited	below	2.267	1.100	339.000	0.063	
	above	3.029	1.337			
JAWS9 fatigue	below	3.733	1.223	217.000	0.402	
	above	3.382	1.349			
JAWS10 inspired	below	1.733	0.884	422.500	<0.001	0.657
	above	3.324	1.273			
JAWS11 relaxed	below	2.200	1.082	421.000	<0.001	0.651
	above	3.500	0.896			
JAWS12 satisfied	below	2.067	1.033	397.000	<0.001	0.557
	above	3.265	1.082			

**Table 4:** Results of the Verbal Fluency Test and the Digit Symbol Test (bold statistically significant features).

	Variable	Before	After	Differences after/before
Verbal Fluency Test	Number of spoken words	14.98	17.02	**4.29**
	Mean time	4.14	3.64	**1.19**
	Popularity of letter	4.08	3.77	2.23
	Fluency coefficient	5.64	5.80	3.98
Digit Symbol Test	Number of total matches	34.47	39.41	6.33
	Number of correct matches	32.41	36.76	5.86
	Digit coefficient	0.92	0.93	0.06

**Table 5:** Features selected using the JMI method.

Signal	Features
BVP	mean (ptest), median (ex3), moda (ex1, ex2), quartile std (ex2), 4th moment (ex1), 5th moment (ex1, vft2 *), total sume (ex1), sume power (ex1, ptest), mean power (ex2, ex3), rms (ex3), entropy (ex3)
EDA	tonicity (vft1), obj (dst1), mean distance regression (dst1), number of GSR (dst1), shift (dst1)

The sign ’*’ marks statistically significant features that were also selected by the JMI criterion.

**Table 6:** Evaluation of the kNN classifier.

	PCA	JMI
Accuracy (ACC)	79.60%	81.63%
Sensitivity (TPR)	88.24%	85.71%
Specificity (TNR)	60.00%	71.43%
Precision (PPV)	0.83	0.88
$F_{1}$	0.86	0.90

## Data Availability

Data not available due to privacy and ethical restrictions.

## Abbreviations

The following abbreviations are used in this manuscript:

ACC X/Y/Z	Accelerometer X/Y/Z
ACC	Accuracy
BVP	Blood Volume Pulse
$c_{v}$	coefficient of variation
D4S	Disc4Spine Project
DL	Deep Learning
EDA	Electroderma Activity
FN	False Negative
FP	False Positive
GSR	Galvanic Skin Response
HR	Heart Rate
JAWS	Job-Related Affective Well–being Scale
JMI	Joint Mutual Information
kNN	k-Nearest Neighbours
ML	Machine Learning
obj	observed data of EDA
PCA	Principal Component Analysis
PPV	precision
TN	True Negative
TNR	True Negative Rate
TP	True Positive
TPR	True Positive Rate
VFT	Verbal Fluency Test
